# Bak instead of Bax plays a key role in metformin-induced apoptosis s in HCT116 cells

**DOI:** 10.1038/s41420-021-00755-y

**Published:** 2021-11-22

**Authors:** Hongce Chen, Beini Sun, Han Sun, Lingjun Xu, Guihao Wu, Zhuang Tu, Xuecheng Cheng, Xuhong Fan, Zihao Mai, Qiling Tang, Xiaoping Wang, Tongsheng Chen

**Affiliations:** 1 MOE Key Laboratory of Laser Life Science & Guangdong Provincial Key Laboratory of Laser Life Science, College of Biophotonics, South China Normal University, 510631 Guangzhou, China; 2 Department of Pain Management, The First Affiliated Hospital of Jinan University, 510632 Guangzhou, China; 3 SCNU Qingyuan Institute of Science and Technology Innovation Co., Ltd., South China Normal University, 511500 Qingyuan, China

**Keywords:** Apoptosis, Pharmacology

## Abstract

Metformin (Met) exhibits anticancer ability in various cancer cell lines. This report aims to explore the exact molecular mechanism of Met-induced apoptosis in HCT116 cells, a human colorectal cancer cell line. Met-induced reactive oxygen species (ROS) increase and ROS-dependent cell death accompanied by plasma membrane blistering, mitochondrial swelling, loss of mitochondrial membrane potential, and release of cytochrome c. Western blotting analysis showed that Met upregulated Bak expression but downregulated Bax expression. Most importantly, silencing Bak instead of Bax inhibited Met-induced loss of mitochondrial membrane potential, indicating the key role of Bak in Met-induced apoptosis. Live-cell fluorescence resonance energy transfer (FRET) analysis showed that Met unlocked the binding of Mcl-1 to Bak, and enhanced the binding of Bim to Bak and subsequent Bak homo-oligomerization. Western blotting analysis showed that Met enhanced AMPK phosphorylation and Bim expression, and compound C, an inhibitor of AMPK, inhibited Met-induced Bim upregulation. Although Met increased the expression of Bcl-xL, overexpression of Bcl-xL did not prevent Met-induced apoptosis. In summary, our data demonstrate for the first time that Met promotes ROS-dependent apoptosis by regulating the Mcl-1-Bim-Bak axis.

## Introduction

Metformin (Met), a biguanide derivative derived from herbal medicine and being used as the first-line oral drug for the treatment of type 2 diabetes, has been demonstrated to prevent a variety of tumors [1]. Numerous preclinical, epidemiological and clinical studies have shown that Met is associated with the reduction of cancer incidence and cancer-related death [1, 2]. Met is thought to have two potential anti-tumor pathways: AMPK-dependent and AMPK-independent pathways. In the AMPK-dependent pathway, Met directly inhibits mitochondrial respiratory chain complex I to limit oxidative phosphorylation, thereby reducing the amount of ATP production, and increasing AMP/ATP ratio and AMPK phosphorylation and activation. AMPK activation inhibits mTOR signaling via phosphorylating and activating p53, leading to autophagy and apoptosis [3, 4]. In the AMPK-independent pathway, Met reduces insulin and insulin-like growth factor-1 [5, 6]. However, the exact mechanism by which Met reduces tumor growth is still unclear.

Colorectal cancer (CRC) ranks third in terms of incidence and the second most common cause of cancer death in the world, characterized by poor prognosis and low 5-year survival rate of 11% [7, 8]. CRC patients are typically treated with conventional cytotoxic chemotherapy, targeted therapy and anti-PD-1 immunotherapy [9, 10]. However, most CRC patients are either inherently insensitive to therapeutic treatment or easy to acquire resistance upon relapse. There is a critical need to develop novel and more effective CRC therapies [11]. Epidemiological studies have found that Met can reduce the incidence, mortality and recurrence risk of CRC in patients with diabetes, thus increasing the survival benefit and the effect of chemotherapy drugs [12, 13]. Experimental studies have shown that Met inhibits proliferation of CRC cells and suppresses intestinal polyp growth in ApcMin^/+^ mice, suggesting that Met may be a novel candidate for CRC [14].

The Bcl-2 family proteins play a key role in regulating apoptosis. Bax and Bak are key effectors to initiate apoptotic pathway. Many kinds of tumors exhibit overexpression of anti-apoptotic proteins (Mcl-1, Bcl-xL, and Bcl-2), near all anti-tumor drugs targeting the Bcl-2 family have focused on the inhibition of Bcl-xL and Bcl-2 [15]. It is reported that Bid most efficiently activates Bak than Bim, whereas Bim exhibits stronger affinity for Bax than Bid [16]. The balance between proapoptotic members and anti-apoptotic components maintains normal physiological activities of cells [17, 18]. Met induces apoptosis by downregulating Bcl-2 and upregulating Bax in ovarian cancer [19, 20], and induces cell death by inhibiting Mcl-1 in hypoglycemia [21]. Further studies are needed to elucidate the precise mechanisms by which Met induces cell death.

This report aims to explore how Met regulate Bcl-2 family proteins to induce apoptosis in HCT116 cells, a kind of CRC cells. Our data firmly proved for the first time that the Mcl-1-Bim-Bak axis mediated the Met-induced apoptosis. Met activates AMPK to increase Bim expression, in turn activates Bak, leading to the release of cytochrome c and subsequent activation of downstream apoptosis signaling pathway. Interestingly, Bcl-xL is not involved in Met-induced apoptosis though Met-increased Bcl-xL expression.

## Results

### Met induces plasma membrane blistering and cytotoxicity

**Fig. 1:**
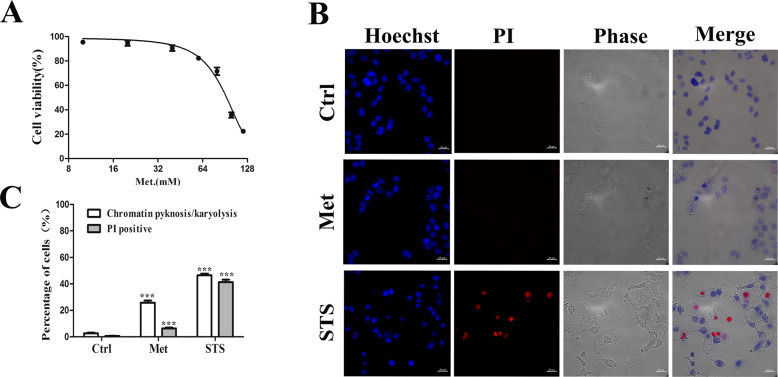
Met induces plasma membrane blistering and cytotoxicity. **A** Met-induced dose-dependent cytotoxicity measured by CCK-8 assay. Cells were seeded into 96-well microplate and incubated with different concentration of Met (0–100 mM) for 24 h. **B** Representative image of cells stained with Hoechst/PI staining after treatment with 60 mM Met for 24 h, or 1 µM STS for 6 h. **C** Statistical percentages of cells with nuclear chromatin pyknosis/karyolysis and PI positive respectively from at least 500 cells. All data are expressed with the mean ± SEM of three independent experiments.***p < 0.001 compared with the control group.

CCK-8 assay showed that treatment with different concentrations (10–100 mM) of Met for 24 h induced dose-dependent cytotoxicity (Fig. 1A). To verify whether Met induces apoptosis and necrosis, we imaged the cells stained by Hoechst/PI, and the STS-treated cells were used as the positive control (Figs. 1B, S1B). Morphological observation showed that Met-treated cells contracted, and more than 50% of the cells showed vesicles over the whole cells surface, but kept their membrane integrity (PI negative), and also exhibited obvious nuclear chromatin pyknosis and karyolysis. After small vesicles become bullae, PI entered the cells (Fig. S1A). The vesicles on the cell membrane surface may be apoptotic bodies. Taken together, Met has potent in vitro anticancer capability against HCT116 cells.

### Met induces ROS-dependent apoptosis in HCT116 cells

**Fig. 2:**
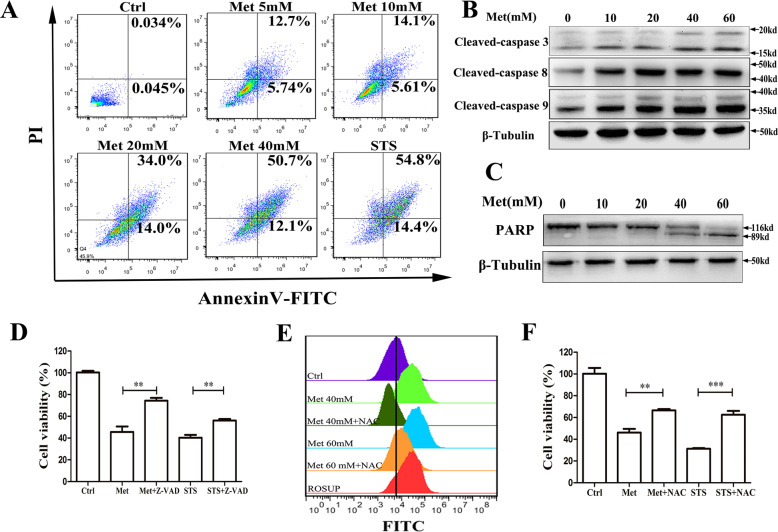
Met induces ROS-dependent apoptosis in HCT116 cells. **A** The percentage of apoptotic cells quantitatively analyzed by using flow cytometry for the cells stained by Annexin-V/PI for at least 10,000 events per sample. **B** Level of cleaved-caspase-3, cleaved-caspase-8, and cleaved-caspase-9 assessed by western blotting for the cells treated with Met for 24 h. **C** Western blotting analysis on the Met-induced PARP cleavage. **D** Z-VAD inhibited Met-induced cytotoxicity. **E** Met or STS induced a significantly increased intracellular ROS. **F** NAC significantly inhibited Met-induced cytotoxicity. All data are expressed with the mean ± SEM of three independent experiments. **p < 0.01 and ***p < 0.001 compared with the control group.

In order to further verify whether Met-induced apoptosis, the percentage of apoptotic cells was quantitatively analyzed by using flow cytometry for the cells stained by Annexin-V/PI. The percentage of apoptotic cells includes early apoptotic cells and late apoptotic cells or necrotic cells. Compared with the control group, a dose-dependent increase of the apoptotic cells was observed after 24 h incubation with different concentrations of Met (Fig. 2A). The caspase activity assay by using Western blotting and enzyme-labeling instrument detection showed that Met-induced activation of caspase-3, caspase-8 and caspase-9 (Figs. 2B, S2), which was further verified by Western blotting analysis on the Met-induced PARP cleavage (Fig. 2C), an important indicator of cell apoptosis and caspase-3 activation [22]. Z-VAD (a broad-spectrum caspase inhibitor) inhibited Met-induced cell death (Fig. 2D), further demonstrating that Met-induced casapses-dependent intrinsic apoptosis. Met or STS induced a significantly increased intracellular reactive oxygen species (ROS) (Fig. 2E), and NAC (reactive oxygen species inhibitor) significantly inhibited Met-induced cell death (Fig. 2F), indicating that Met-induced ROS production and ROS-dependent apoptosis.

### Bak instead of Bax plays a key role in Met-induced apoptosis

**Fig. 3:**
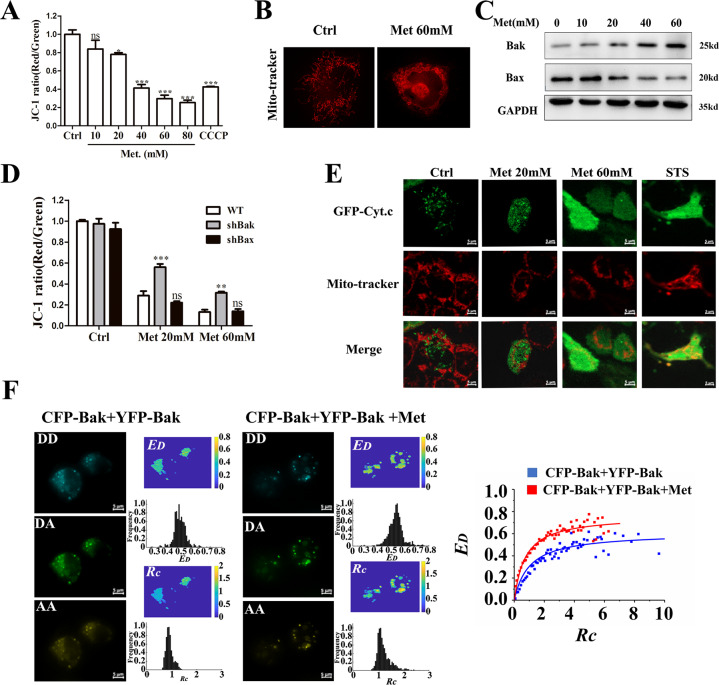
Bak instead of Bax plays a key role in Met-induced apoptosis. **A** Met-induced dose-dependent loss of mitochondrial membrane potential (*n* = 60, ***p < 0.001 compared with the control group). **B** Met-induced mitochondrial swelling. Cells were stained with Mito-tracker Deep Red staining after treatment with 60 mM Met for 24 h. **C** Met upregulated Bak and downregulated Bax by western blotting analysis. Cells were treated with Met for 24 h. **D** Silencing Bak instead of Bax inhibited Met-induced loss of mitochondrial membrane potential (*n* = 60, **p < 0.01; ***p < 0.001 compared with the WT group). **E** Met-induced release of cytochrome c from mitochondria. Cells expressing GFP-cytochrome c were imaged. **F** Quantitative FRET measurements in living cells co-expressing CFP-Bak and YFP-Bak and the corrresponding *ED-Rc* plot from at least 108 cells. All data are expressed with the mean ± SEM of three independent experiments.

We used JC-1 probe to detect whether Met-induced loss of mitochondrial membrane potential (Fig. 3A). Fluorescence microscope imaging revealed that Met-induced loss of mitochondrial membrane potential in concentration-dependent fashion. Microscopic imaging also showed that Met caused mitochondrial swelling (Fig. 3B), further indicating that Met impaired mitochondrial function. Bax and Bak, as the main effectors of Bcl-2 family proteins, play a key role in the intrinsic apoptosis pathway [23]. Western blotting analysis showed that Met upregulated Bak expression but downregulated Bax expression in HCT116 cells (Fig. 3C). Moreover, silencing Bak instead of Bax inhibited Met-induced loss of mitochondrial membrane potential (Fig. 3D), indicating that Bak instead of Bax plays a key role in Met-induced apoptosis. We next assessed the distribution of cytochrome c in single living cells expressing GFP-cytochrome c by using fluorescence microscopic imaging (Fig. 3E). In control cells, cytochrome c mainly concentrated in mitochondria. In Met-treated cells, cytochrome c was released from mitochondria and distributed uniformly in the cytoplasm.

Next, quantitative fluorescence resonance energy transfer (FRET) measurements were performed in living cells co-expressing CFP-Bak and YFP-Bak to explore whether Met-induced Bak homo-oligomerization. Figure 3F shows the fluorescence images of representative cells co-expressing CFP-Bak and YFP-Bak in the absence (control) or presence of Met, and the corresponding pixel-to-pixel pseudo-color *E*_*D*_ and *Rc* images as well as their histograms. The two *E*_*D*_–*R*_*C*_ binding curves from at least 108 cells were saturable when *Rc* was larger than 4, and the 0.765 of *E*_*D*max_ in Met-treated cells was larger than the 0.609 of *E*_*D*max_ in control cells (Fig. 3F), indicating that Met-increased Bak homo-oligomerization.

### Met induces apoptosis by relieving the binding of Mcl-1 to Bak

**Fig. 4:**
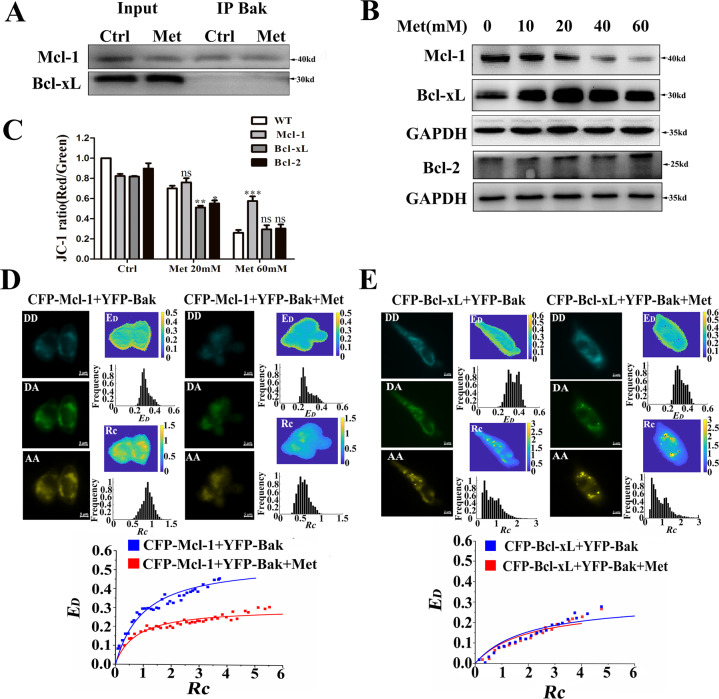
Met induces apoptosis by relieving the binding of Mcl-1 to Bak. **A** Co-immunoprecipitation analysis on the binding of Mcl-1/Bcl-xL to Bak. **B** Western blotting analysis on the Mcl-1 and Bcl-xL expression after Met treatment for 24 h. **C** Overexpression Mcl-1 instead of Bcl-2 or Bcl-xL inhibited Met-induced loss of mitochondrial membrane potential (*n* = 53, ***p < 0.001 compared with the WT group). **D** Quantitative FRET measurements for living cells co-expressing CFP-Mcl-1 and YFP-Bak, and the corresponding *E*_*D*_*–Rc* plot from at least 109 cells. **E** Quantitative FRET measurements for living cells co-expressing CFP-Bcl-xL and YFP-Bak, and the corresponding *E*_*D*_*–Rc* plot from at least 99 cells. All data are expressed with the mean ± SEM of three independent experiments.

Anti­apoptotic proteins (Bcl-2, Bcl-xL, Mcl-1) inhibit apoptosis by binding to proapoptotic proteins [16]. HCT116 cells exhibit high expression of Mcl-1 and Bcl-xL [15]. Co-immunoprecipitation analysis showed that Mcl-1 strongly bound to Bak, while Bcl-xL weakly bound to Bak (Fig. 4A). Western blotting analysis showed that Met decreased Mcl-1 expression, but elevated Bcl-xL expression (Fig. 4B). However, overexpression of Mcl-1 instead of Bcl-2/Bcl-xL significantly inhibited Met-induced loss of mitochondrial membrane potential (Fig. 4C), indicating that Mcl-1 instead of Bcl-xL plays a key inhibitory role in Met-induced apoptosis.

Quantitative FRET measurements were performed for the living cells co-expressing CFP-Mcl-1 and YFP-Bak (Fig. 4D). Figure 4D also shows the corresponding *E*_*D*_–*Rc* plots from 109 cells. The two binding curves were saturable when *Rc* was larger than 2, and the 0.306 of *E*_*D*max_ in Met-treated cells was smaller than the 0.470 of *E*_*D*max_ in control cells, indicating that Met unlocked the binding of Mcl-1 to Bak. However, the *E*_*D*max_ (0.311, *n* = 99) between CFP-Bcl-xL and YFP-Bak in control cells was similar to *E*_*D*max_ (0.295, *n* = 104) in Met-treated cells (Fig. 4E), indicating that Met has no effect on the binding of Bcl-xL to Bak.

### Bim is the key BH3-only protein to initiate Met-induced apoptosis

**Fig. 5:**
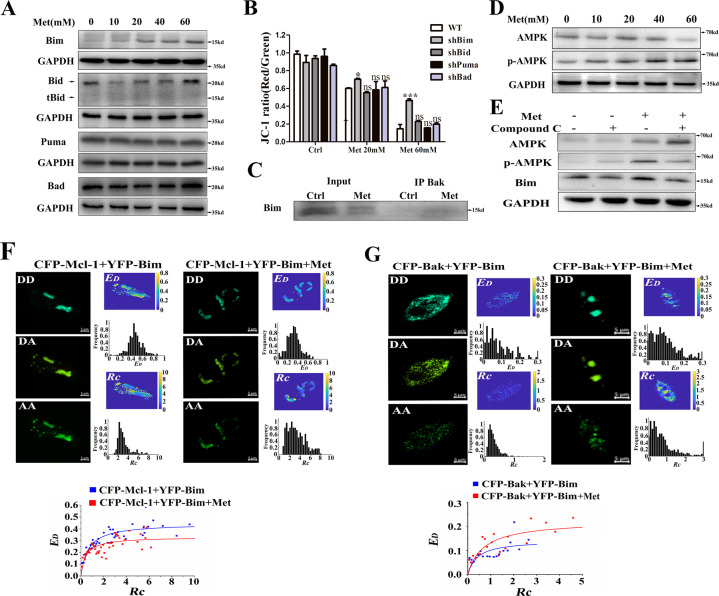
Bim is the key BH3-only protein to initiate Met-induced apoptosis. **A** Western blotting analysis on Puma, Bim, Bid and Bad expression after Met treatment for 24 h. **B** Effects of silencing Puma, Bim, Bid, and Bad respectively on the Met-induced loss of mitochondrial membrane potential (*n* = 38, *p < 0.05 and ***p < 0.001 com*p*ared with the WT group). **C** Co-immunoprecipitation analysis on the binding of Bim to Bak. **D** Met enhanced the AMPK phosphorylation. **E** Compound C inhibited Met-induced AMPK phosphorylation and Bim expression. **F** Quantitative FRET measurements for living cells co-expressing CFP-Mcl-1 and YFP-Bim, and the corresponding *E*_*D*_*–Rc* plot from at least 100 cells. **G** Quantitative FRET measurements for living cells co-expressing CFP-Bak and YFP-Bim, and the corresponding *E*_*D*_*–Rc* plot from at least 90 cells. All data are expressed with the mean ± SEM of three independent experiments.

BH3-only proteins including Bim, tBid, and Puma can directly activate Bak [16]. Western blotting analysis showed that Met promoted the expression of Bim, but did not cleave Bid into tBid and did not influence the expression of Puma and Bad (Fig. 5A). Only silencing Bim significantly inhibited Met-induced loss of mitochondrial membrane potential (Fig. 5B), suggesting that Bim plays an important role in the Met-induced apoptosis. Co-immunoprecipitation analysis showed that Bim weakly bound to Bak in normal cells, and Met promoted the binding of Bim to Bak (Fig. 5C). Western blotting analysis showed that Met enhanced the AMPK phosphorylation (Fig. 5D), and compound C inhibited Met-induced Bim upregulation (Fig. 5E), indicating that Met activates AMPK to upregulates Bim.

Next, in order to evaluate the effect of Met on the binding of Mcl-1 to Bim, quantitative FRET measurements were used to measure the *E*_*D*_ between CFP-Mcl-1 and YFP-Bim in 101 control cells and 106 Met-treated cells, respectively (Fig. 5F). The two binding curves were saturable when *Rc* was larger than 2, and the 0.3316 of *E*_*D*max_ in Met-treated cells was smaller than the 0.4456 of *E*_*D*max_ in control cells, indicating that Met unlocked the binding of Mcl-1 to Bim. Similarly, quantitative FRET measurements were performed for the living cells co-expressing CFP-Bak and YFP-Bim, and Fig. 5G shows the corresponding *E*_*D*_–*Rc* plots from 92 control cells and 91 Met-treated cells. The two binding curves were saturable when *Rc* was larger than 1.0, and the 0.224 of *E*_*D*max_ between CFP-Bak and YFP-Bim in Met-treated cells was smaller than the 0.142 of *E*_*D*max_ in control cells, indicating that Met promoted the binding of Bim to Bak, which may activate Bak.

## Discussion

In this report, we dissected the molecular mechanism of Met-induced apoptosis in HCT116 cells. To our best knowledge, this is the first published report demonstrating that Met induces caspase- and ROS-dependent apoptosis dominantly via a Bak-mediated intrinsic pathway, in which BH3 only protein Bim, as a key initiator, unlocks the binding of Mcl-1 to Bak and then directly activates Bak.

Our observations that silencing Bak but not Bax by shRNA significantly prevented Met-induced loss of mitochondrial membrane potential (Fig. 3D) demonstrate that Bak, but not Bax, plays a key role in Met-induced apoptosis, which is further verified by Met-increased Bak homo-oligomerization (Fig. 3F). Inactive Bax is usually distributed in the cytoplasm and translocates to the mitochondria when it receives an apoptotic signal, while Bak is located in the mitochondrial membrane [24]. Microscopic imaging of cells expressing CFP-Bax showed that Met did not induce Bax translocation from cytosol to mitochondrion (data not shown), which also suggests that Bax is not involved in Met-induced apoptosis. It is generally considered that Bax plays a major role in the intrinsic apoptotic pathway, and Bak is an auxiliary role [25, 26]. However, recent publications reported that Bak plays a key role in apoptosis induced by artesunate, actinomycin D and tumor necrosis factor-related apoptosis-inducing ligand (TRAIL), respectively [27, 28]. We found that Met-induced Bax upregulation and Bcl-xL down-regulation in MCF-7 and A549 cells (Fig. S3) support previous notion that Bax and Bcl-xL plays an important role in metformin-induced apoptosis [29, 30]. However, our observations that Bak instead of Bax plays a key role in Met-induced apoptosis in HCT116 cells.

The findings that overexpression of Mcl-1 instead of Bcl-2 or Bcl-xL inhibited Met-induced loss of mitochondrial membrane potential (Fig. 4C) demonstrate that Mcl-1 plays an important role in inhibiting Met-induced apoptosis, which is further verified by the findings that Met unlocked the binding of Bak to Mcl-1 but did not affect the binding of Bak to Bcl-xL (Fig. 4D, E). Bcl-xL and Mcl-1 can efficiently inhibit Bak activation [16, 31]. The dynamic balance between Mcl-1/Bcl-xL and Bak will be broken by apoptosis stimuli [16]. However, in some cases, Mcl-1 and Bcl-xL do not work at the same time [16, 21]. For example, Met-induced apoptosis in OVCAR-3 and OVCAR-4 cells by downregulating Bcl-2 and Bcl-xL [20]. In MYC-dependent apoptosis, Met acted by inhibiting Bcl-2 and Bcl-xL instead of Mcl-1 [32]. However, Met promoted cell death by downregulating the expression of Mcl-1 under low glucose conditions [21]. Our live-cell FRET imaging that Met unlocked the binding of Mcl-1 to Bak (Fig. 4D) and Met-increased Bak homo-oligomerization (Fig. 3F) firmly demonstrate that Met induces Bak-mediated apoptosis by inhibiting Mcl-1 in HCT116 cells.

Our observations that silencing Bim instead of Bid, Puma and Bad inhibited Met-induced loss of mitochondrial membrane potential (Fig. 5) demonstrate that Bim is the key BH3-only protein to initiate Met-induced apoptosis, which is further verified by the findings that Met unlocked the binding of Mcl-1 to Bim and enhanced the binding of Bim to Bak (Fig. 4D,E). Bid, Bim and Puma are capable of directly activating Bax/Bak [33]. Puma exhibits a weak direct interaction with Bax/Bak compared with Bim and tBid [34, 35]. tBid is generally considered to be has stronger proapoptotic ability than Bim and plays an important role in apoptosis induced by many kinds of stimuli. The active caspase-8 cleaves Bid into tBid to activate Bax/Bak [36, 37]. Although Met-induced caspase-8 activation (Fig. 3B), Met did not cleave Bid into tBid (Fig. 5A), indicating that Bid may not be involved in Met-induced apoptosis, which is further verified by the finding that silencing Bid did not prevent Met-induced apoptosis. In addition, several studies reported that TRAIL induced caspase-8 activation and Bid cleavage, but tBid was unable to induce Bak oligomerization and MOMP [38]. Our findings that Met enhanced AMPK phosphorylation and compound C inhibited Met-induced Bim upregulation (Fig. 5D, E) support previous notion that AMPK plays a crucial role in Bim upregulation [39]. Our data that Met not only decreases Mcl-1 expression (Fig. 4B) and relieves the binding of Mcl-1 to Bim (Fig. 5F) but also increases Bim expression (Fig. 5A) and enhances the binding of Bim to Bak (Fig. 5G) firmly demonstrate the key role of Bim in Met-induced apoptosis.

**Fig. 6:**
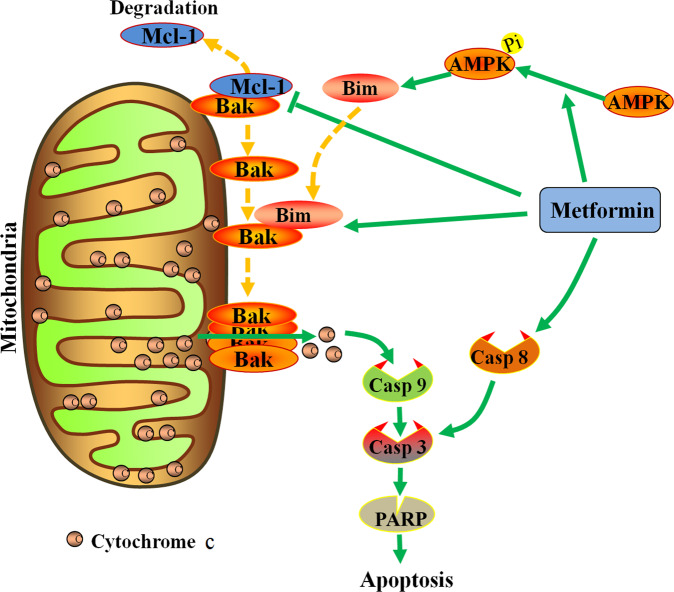
Schematic diagram of the molecular mechanism of Met against HCT116 cells. Met induces the phosphorylation and activation of AMPK to up-regulate Bim. Meanwhile, Met unlocks the binding of Mcl-1 to Bak, and promotes the binding of Bim to Bak, which leads to Bak activation and homo-oligomerization, leading to cytochrome c release and caspase activation of downstream apoptosis signaling pathway.

Based on our data, we summarize the Met-induced apoptotic pathway in HCT116 cells (Fig. 6). Met induces Bak-mediated apoptosis in ROS- and caspase-dependent fashion. Met induces the phosphorylation and activation of AMPK to upregulate Bim. Meanwhile, Met unlocks the binding of Mcl-1 to Bak, and promotes the binding of Bim to Bak, which leads to Bak activation and homo-oligomerization, leading to MOMP and subsequent cytochrome c release and caspase activation. However, how Bim participates in the molecular mechanism by which Met inhibits the interaction between Bak and Mcl-1is unknown, which will be the task of future investigations.

## Materials and methods

### Materials

Met was purchased from Solarbio (Beijing, China). Staurosporine (STS), Caspase-3/8/9 Assay Kit, ROS Assay Kit and JC-1 Assay Kit were provided by Beyotime (Shanghai, China). Anti-PARP (9532), anti-Bak (12105), anti-Bax (5023), anti-Bcl-xL (2764), anti-Mcl-1 (94296), anti-Bim (2933), anti-Bad (9268), anti-Puma (98672), anti-AMPK (5831), anti-p-AMPK (8208), anti-Cleaved Caspase-3 (9664), anti-Cleaved Caspase-8 (8592), anti-Caspase-9 (9502) Rabbit monoclonal antibody, and anti-Bid (8762) Mouse monoclonal antibody were purchased from Cell Signaling Technology (Danvers, MA, USA). Anti-GAPDH (sc-47724) Mouse monoclonal antibody was obtained from Santa Cruz (Texas, USA). Luciferase Mycoplasma Detection Kit and anti-β-tubulin (HC101) Mouse monoclonal antibody were purchased from Transgen Biotech (Beijing, China). Turbofect™ transfection regent and Mito-tracker Deep Red dye were obtained from ThermoFisher Scientific (Massachusetts, USA). Cell counting Kit-8 (CCK-8) and Alexa Fluor^®^ 488 annexin V/Dead Cell Apoptosis Kit were purchased from Dojindo (Kyushu, Japan). Acetylcysteien (NAC), Z-VAD-(OMe)-FMK (Z-VAD) and Compound C were purchased from MedChemExpress (New Jersey, USA). Hoechst 33258 dye and PI dye were obtained from G-clone (Beijing, China).

### Constructs

The CFP-YFP dimers including YFP-G10-CFP and YFP-G40-CFP were kindly provided by Christian Wahl-Schott [40]. Plasmids encoding the CFP-Bcl-xL was kindly supplied by A. P. Gilmore [41]. pTOPO-Mcl-1 (#21065), ECFP-Bak (#31501) plasmids were purchased from Addgene Company. YFP-Bim was synthesized by Gene Create Company. YFP-Bak, CFP-Mcl-1 plasmids and sh Bax, shPuma, shBad were constructed as previously described [27, 42–45]. The silencing plasmids of Bak, Bim and Bid (#p31235, #p31337, #p31234) were designed and synthesized by Miaoling Biotech Company (Wuhan, China). Sequences for shRNA were: Bak shRNA 1#, 5′-GTTTGTGGTACGAAGATTCAAGAGATCTTCGTACCACAAACTGGCCTTTTTTG-3′; Bak shRNA 2#, 5′-GTTTGTGGTACGAAGATTCAAGAGATCTTCGTACCACAAACTGGCCTTTTTTG-3′, Bak shRNA 3#,5′-GTTTGTGGTACGAAGATTCAAGAGATCTTCG;TACCACAAACTGGCCTTTTTTG-3′; Bim shRNA 1#,5′-GCAACCTTCTGATGTAAGTTCTTCAAGAGAGAACTTACATCAGAAGGTTGCTT-3′; Bim shRNA 2#,5′-GGCCTTCAACCACTATCTCAGTTCAAGAGACTGAGATAGTGGTTGAAGGCCTT-3′; Bim shRNA 3#,5′-GCTACCTCCCTACAGACAGATTCAAGAGATCTGTCTGTAGGGAGGTAGCC-3′. All constructs used in this work were confirmed by DNA sequencing and enzyme digestion diagnosis.

### Cell lines and cell culture

The HCT116 cells, MCF-7 cells, and A549 cells were obtained from the tumor cell bank of the Chinese Academy of Medical Science. The cells were cultured in Medium DMEM containing 10% fetal bovine serum, 100 U/mL penicillin G and 100 µg/mL streptomycin in a humidified atmosphere of 5% CO_2_ at 37 °C. All experiments were performed during the exponential phase of cell growth. Stocks of authenticated cell lines were cryopreserved, all cell lines were in early passages and maintained in culture for <3 months, tested regularly for mycoplasma contamination using luciferase mycoplasma detection kit.

### Cell viability assay

The inhibitory effect of Met on the viability of HCT116 cells were measured by the CCK-8 assay according to the protocol described by the manufacturer. In brief, cells were seeded in 96-well plates at a density of 1.0 × 10^5^ cells/well with a volume of 100 μL and grown at 37 °C for 24 h, then treated with different concentrations of Met for 24 h. Subsequently, 10 μL of CCK-8 solution was added to each well and the plates were incubated at 37 °C for 1 h, after which the optical density of each well was determined at 570 nm using a microplate reader. All the experiments were repeated three times independently. The half maximal inhibitory concentration (IC50) values of Met in HCT116 cells were calculated.

### Characterization of cell death

The morphology of nucleus was analyzed by Hoechst /PI double staining. In brief, cells were grown on the confocal dishes for 24 h. After being treated with indicated treatments, the cells were incubated with 20 µg/mL Hoechst for 30 min and then with 10 µg/mL PI for 15 min at 37 °C in the dark. Then, the cells were washed once with PBS and observed with fluorescence microscope.

### Flow cytometry

Identification of apoptotic cells was achieved by Annexin V-FITC/PI staining. HCT116 cells (2.0 × 10^5^ cells/mL) were seeded in six-well plates and treated with different concentrations of Met for 24 h. Annexin V-FITC/PI staining was performed according to the instructions provided by the manufacturer. 1.0 × 10^6^ cells per sample was collected by 0.2% Trypsin, washed twice with PBS, cells were resuspended with 1× binding buffer at a density of 1.0 × 10^6^ cells/mL. Annexin V-FITC and PI (5 µL) were added to cell suspensions (100 µL) and further incubated for 15 min at room temperature in the dark. Stained cells were diluted with 1× binding buffer and immediately analyzed by flow cytometry. All experiments were repeated three times.

### Caspase activity assay

The HCT116 cells were placed in 6-well plates at 2 × 10^5^ cells/well. According to the manufacturer’s protocol, treated cells were lysed with lysis buffer for 15 min on ice following washing with cold PBS, the supernatants collected and protein concentration determined by BCA protein assay. Incubate the mixture composed of 10 µL of cell lysate, 80 µL of reaction buffer and 10 µL of 2 mM caspase-3 substrate in 96-well microtiter plates at 37 °C for 4 h, and the activity of caspase-3 were quantified in the samples with enzyme-labeling instrument at an absorbance of 405 nm. Relative caspase activity was calculated as the emission ratio between the treated cells to the untreated cells.

### Detection of intracellular ROS

Intracellular ROS was detected by means of an oxidation-sensitive fluorescent probe (DCFH-DA). After treatment with Met for 24 h, cells were washed twice in phosphate-buffered saline (PBS), then incubated with 10 µM/L DCFH-DA at 37 °C for 30 min according to the manufacturer’s instructions. DCFH-DA was deacetylated intracellularly by nonspecific esterase, which was further oxidized by ROS to the fluorescent compound 2,7-dichlorofluorescein (DCF). DCF fluorescence was detected by flow cytometer.

### Western blotting analysis

For the extraction of whole proteins, cells were collected and washed twice with pre-cold PBS. Cells were then treated with RIPA lysis buffer containing protease inhibitors to obtain cell lysates. Next, the lysates were sonicated and centrifuged at 13,000 rpm for 10 min. Supernatant was recovered and quantified by the BCA protein assay, and stored at −20 °C. The proteins were separated by 12% SDS-PAGE, electrophoretically transferred onto 0.22 μm polyvinylidene difluoride membranes. After blocking with 5% skimmed milk at room temperature for 2 h, the membranes was incubated overnight at 4 °C with antibodies: rabbit anti-human Bak antibody (1: 1 000). After washing three times with TBST, the membranes were incubated with HRP-labeled goat anti-rabbit polyclonal antibody (1: 10,000) at room temperature for 2 h. After washing with TBST buffer, the immunoreactive bands were visualized by enhanced chemiluminescence chemistry substrate.

### Co-Immunoprecipitation

HCT116 cells were lysed in RIPA buffer with protease inhibitor for 5 min, centrifuged at 13,000 rpm for 10 min at 4 °C and the supernatants were collected of total lysates, then immunoprecipitated with anti-Bak antibody at room temperature for 2 h. Collected the Protein-A/G magnetic beads, the beads were washed with RIPA buffer three times. All protein samples were boiled for 5 min in standard loading buffer with 20% loading buffer before western blotting detection.

### Quantitative FRET imaging

Quantitative FRET imaging was performed on a fluorescence microscope (Axio Observer 7, Carl Zeiss, Oberkochen, Germany) as previously described [17]. Donor-centric FRET efficiency (*E*_*D*_) and acceptor–donor ratio (*Rc*) were measured by using FRET method just as described previously [46, 47].

### *E*_*D*_ saturation assay

Cells were co-transfected with 200 ng of plasmid encoding CFP-tagged protein and various concentrations (200, 400, 600, and 800 ng, respectively) of plasmid encoding YFP-tagged protein. In general, only the cells with fluorescence signals at least three times higher than backgrounds and <60,000 (the saturation value of fluorescence signal is 65,535) were chosen for analysis. *E*_*D*_ values were distributed into bins of different sizes according to concentration ratio of total acceptor-to-donor (*Rc*) and plotted against Rc. The saturation binding curves were fitted using Origin with the function: *E*_*D*_ = *E*_*Dmax*_ *×* *Rc/(K*_*d*_ + *Rc)*. *E*_*Dma*x_ is the maximum *E*_*D*_ corresponding to saturation of donor binding sites by an acceptor and *K*_*d*_ is the relative equilibrium dissociation constant [43, 48].

### Statistical analysis

The *p* values for datasets were analyzed by unpaired Student’s *T* test, *p* < 0.05 was defined as statistical significance. Statistical and graphic analyses were done using the software Graph Pad Prism 5 and Origin 8.0.

## Supplementary information

Supplementary Figure Legends

Supplemental Figure 1

Supplemental Figure 2

Supplemental Figure 3

## Data Availability

The datasets used and/or analyzed during the current study are available from the corresponding author on reasonable request.
