# Supplementary material for: Bak instead of Bax plays a key role in metformin-induced apoptosis s in HCT116 cells
Source: Cell Death Discov. 2021 Nov 22;7:363. doi: 10.1038/s41420-021-00755-y (PMC8608863; doi:10.1038/s41420-021-00755-y)
Supplement: Supplementary file 1 — Supplementary Figure Legends [file 41420_2021_755_MOESM1_ESM.docx]

**Supplemental Fig.1** Met induces plasma membrane blistering and cytotoxicity. **A** Statistical percentages of vesicular cells and bullous cells at the indicated times after Met or STS treatment from at least 500 cells. Blue arrows indicate vesicles, red arrow indicates bullae. **B** Representative bright field images of cells treated with 60 mM Met for 24 h, the enlarged drawings show the bubbling cells. All data are expressed with the mean ± SEM of three independent experiments. ****p*< 0.001 compared with the control group.

**Supplemental Fig.2** Met induced activation of caspase-3, caspase-8 and caspase-9. All data are expressed with the mean ± SEM of three independent experiments. ***p*< 0.01 and ****p*< 0.001 compared with the control group.

**Supplemental Fig.3** Western blotting analysis on Bak, Bax, Mcl-1 and Bcl-xL expression in MCF-7 and A549 cells treated with Met for 24 h.
