# Supplementary figures and images for: Bak instead of Bax plays a key role in metformin-induced apoptosis s in HCT116 cells
Source: Cell Death Discov. 2021 Nov 22;7:363. doi: 10.1038/s41420-021-00755-y (PMC8608863; doi:10.1038/s41420-021-00755-y)

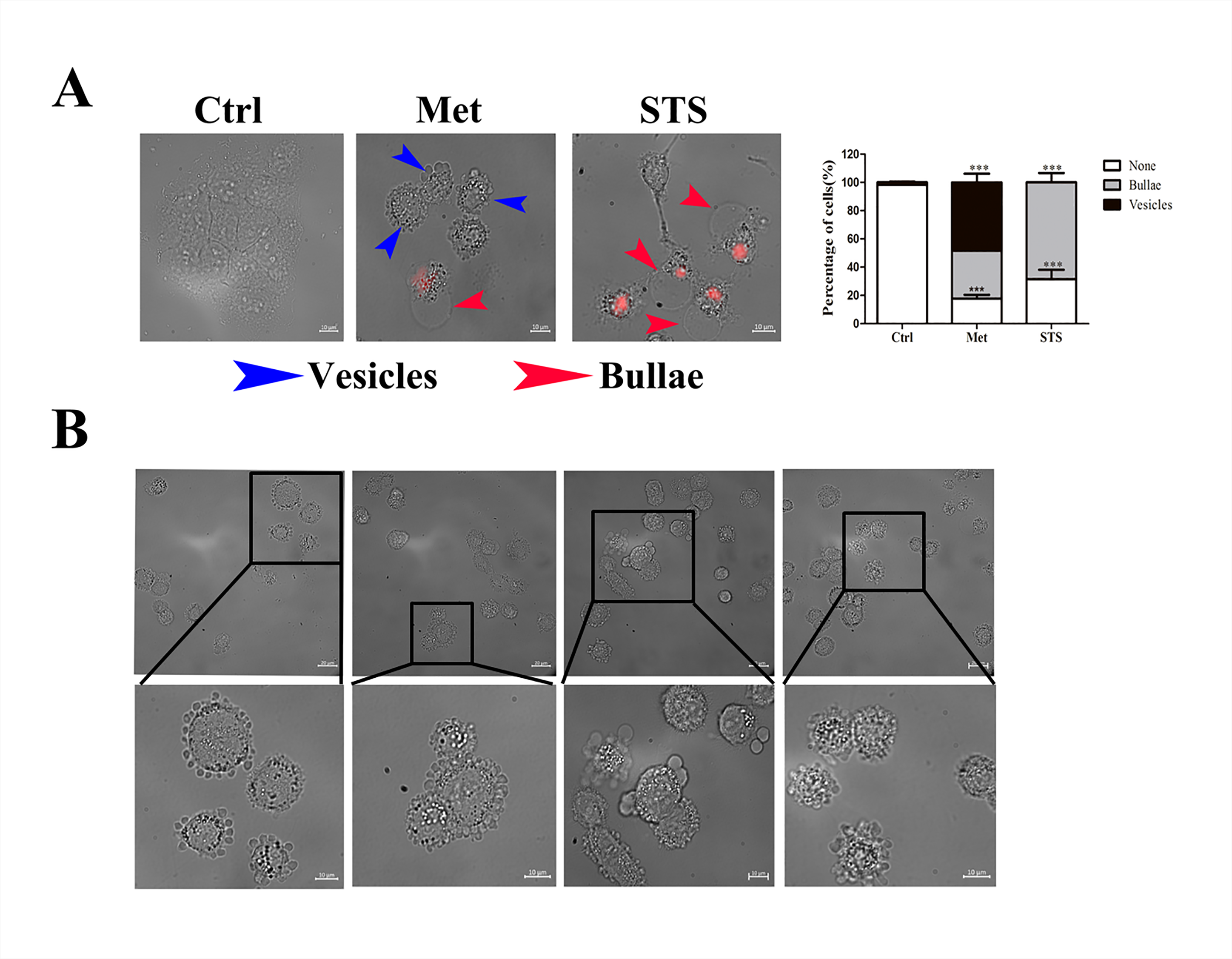

Supplement: Supplementary file 2 — Supplemental Figure 1 [file 41420_2021_755_MOESM2_ESM.tif]

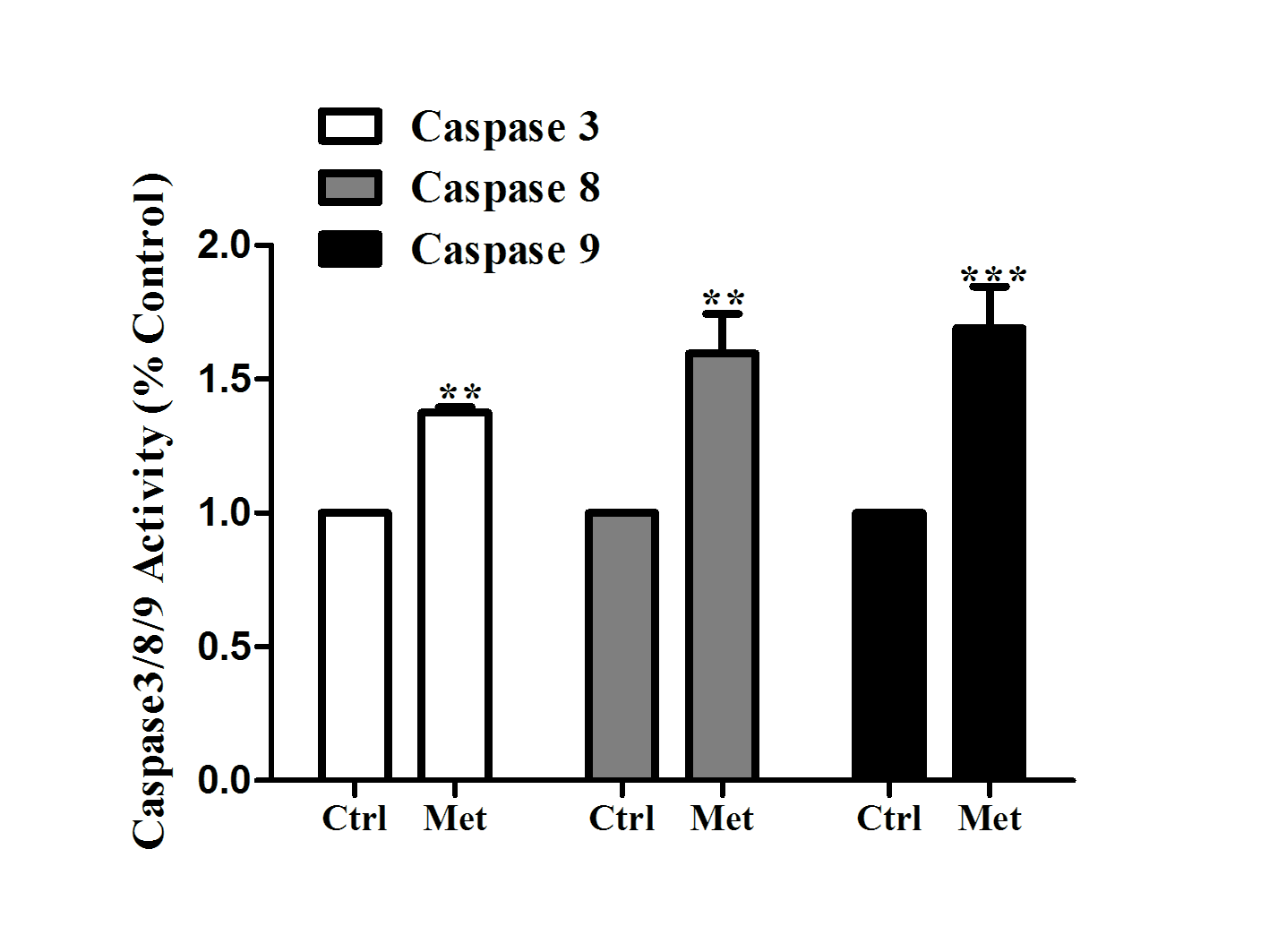

Supplement: Supplementary file 3 — Supplemental Figure 2 [file 41420_2021_755_MOESM3_ESM.tif]

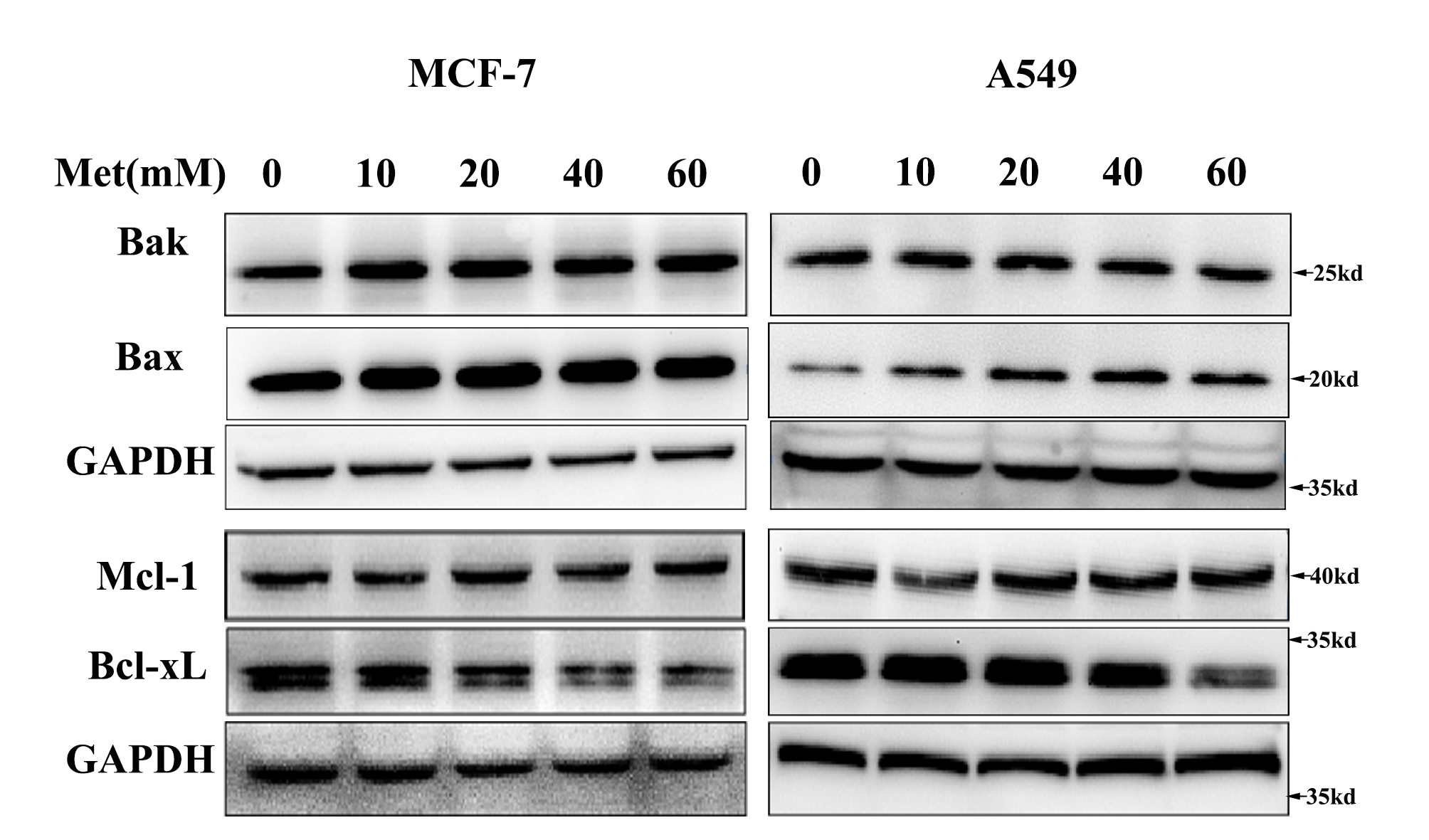

Supplement: Supplementary file 4 — Supplemental Figure 3 [file 41420_2021_755_MOESM4_ESM.tif]
